# Supplementary material for: Chromothripsis during telomere crisis is independent of NHEJ, and consistent with a replicative origin
Source: Genome Res. 2019 May;29(5):737–49. doi: 10.1101/gr.240705.118 (PMC6499312; doi:10.1101/gr.240705.118)
Supplement: Supplemental Material [file supp_gr.240705.118_Supplemental_file_1.zip › contigs/annotated_contigs/DB37/contig.2.DB37_length_433_mean_cov_5.88914549654.docx]

**DB37_length_433_mean_cov_5.88914549654**

```
GTGTTTCACTCATTTTTAAAATTGACTTTTTGACTTCTTCCTGTAGAGTTTAGAGAGTTCTTTCTGCAGCTGGATATAAGTCCTTTGTT
 >chr15:66511639-66511738 - E=5e-48
GGCTATGTGA|CTGTAT|GCAGAACTGAGGATGCTCTGTTGGATGGCTCTTGTCACTCTACAGGAATGCATGAAAGAGAGCCACGATCT
                 >chr15:66584037-66584365 - E=4e-186
CCCACTCATTGTACTTCCATGTCAAGTTACCCTGCAGTGTGAGCTGATGTGAATGAGATCTGAGGTGGAATTGCACGAATTAGGAAAAC

AGCACAGAGCTGGCCTGTAAGGCAACCAGCAGTGCAGGAGGCTGTCCCTAATTCAGGAGGTCAGTTCTGGACAGCCCAGAGGACAGTCA

AGTTGCCAGATTTGTCCCCTACATAAATATGTTCCAGGCAGCTGACCACAGTTCCCTTCAAAGCTCCCTCACGTTCATT
```
